# Supplementary material for: Digital health for chronic disease management: An exploratory method to investigating technology adoption potential
Source: PLoS One. 2023 Apr 13;18(4):e0284477. doi: 10.1371/journal.pone.0284477 (PMC10101441; doi:10.1371/journal.pone.0284477)
Supplement: S2 File — (DOCX) [file pone.0284477.s002.docx]

Supplementary File 2: Cluster comparison

| **Number** | **772** | **88** | **130** |
| --- | --- | --- | --- |
| Male sex | 195 (25.3) | 17 (19.3) | 42 (32.3) |
| Paper questionnaire participant | 93 (12.0) | 9 (10.2) | 56 (43.1) |
| Year of birth; N, median [interquartile range] | 761, 1,971 [1,963; 1,980] | 85, 1,974 [1,967; 1,983] | 128, 1,960 [1,952.5; 1,966] |
| Year of diagnosis; N, median [interquartile range] | 754, 2,010.5 [2,003; 2,016] | 83, 2,012 [2,003; 2,016] | 124, 2,001.5 [1,996; 2,010] |
| MS type |  |  |  |
| Unknown MS type | 25 (3.2) | 7 (8) | 4 (3.1) |
| Clinically Isolated Syndrome (CIS) | 14 (1.8) | 1 (1.1) | 0 (0.0) |
| Primary progressive MS (PPMS) | 78 (10.1) | 8 (9.1) | 22 (16.9) |
| Relapsing remitting MS (RRMS) | 517 (67.0) | 62 (70.5) | 54 (41.5) |
| Secondary progressive MS (SPMS) | 118 (15.3) | 9 (10.2) | 44 (33.8) |
| Transitional phase | 20 (2.6) | 1 (1.1) | 6 (4.6) |
| Unknown MS types | 20 (2.6) | 6 (6.8) | 4 (3.1) |
| EDSS proxy measure |  |  |  |
| SRDSS 0-3.5 | 545/735 (74.1) | 56/80 (70.0) | 53/122 (43.4) |
| SRDSS 4-6.5 | 139/735 (18.9) | 14/80 (17.5) | 43/122 (35.2) |
| SRDSS 7 and higher | 51/735 (6.9) | 10/80 (12.5) | 26/122 (21.3) |
| Occurrence of self-reported symptoms |  |  |  |
| Affective disorder | 63/694 (9.1) | 8/73 (11.0) | 10/122 (8.2) |
| Balance problems | 236/750 (31.5) | 38/81 (46.9) | 55/125 (44.0) |
| Bladder problems | 205/750 (27.3) | 28/81 (34.6) | 44/125 (35.2) |
| Concentration problems | 165/694 (23.8) | 28/73 (38.4) | 39/122 (32.0) |
| Depression | 74/750 (9.9) | 18/81 (22.2) | 12/125 (9.6) |
| Dizziness | 126/750 (16.8) | 23/81 (28.4) | 22/125 (17.6) |
| Dysphagia | 54/750 (7.2) | 7/81 (8.6) | 16/125 (12.8) |
| Epilepsy | 2/750 (0.3) | 2/81 (2.5) | 1/125 (0.8) |
| Fatigue | 338/750 (45.1) | 51/81 (63.0) | 71/125 (56.8) |
| Gait problems | 227/750 (30.3) | 27/81 (33.3) | 67/125 (53.6) |
| Gastrointestinal problems | 139/750 (18.5) | 20/81 (24.7) | 36/125 (28.8) |
| Memory problems | 122/750 (16.3) | 22/81 (27.2) | 24/125 (19.2) |
| Other symptoms | 14/750 (1.9) | 1/81 (1.2) | 1/125 (0.8) |
| Pain | 187/750 (24.9) | 34/81 (42.0) | 41/125 (32.8) |
| Paralysis | 65/750 (8.7) | 13/81 (16.0) | 27/125 (21.6) |
| Paresthesia | 301/750 (40.1) | 50/81 (61.7) | 54/125 (43.2) |
| Sexual problems | 87/750 (11.6) | 17/81 (21.0) | 24/125 (19.2) |
| Spasms | 186/750 (24.8) | 25/81 (30.9) | 54/125 (43.2) |
| Spatial disorientation | 26/694 (3.7) | 2/73 (2.7) | 5/122 (4.1) |
| Tics | 63/750 (8.4) | 9/81 (11.1) | 18/125 (14.4) |
| Tremors | 56/750 (7.5) | 8/81 (9.9) | 11/125 (8.8) |
| Visual disturbances | 103/750 (13.7) | 22/81 (27.2) | 34/125 (27.2) |
| Weakness | 201/750 (26.8) | 36/81 (44.4) | 56/125 (44.8) |
| Disease-modifying treatment (DMT) |  |  |  |
| Never received DMTs | 239/750 (31.9) | 25/81 (30.9) | 71/125 (56.8) |
| Has received one DMT | 504/750 (67.2) | 53/81 (65.4) | 53/125 (42.4) |
| Has received more than one DMT | 7/750 (0.9) | 3/81 (3.7) | 1/125 (0.8) |
| Currently employed | 486/736 (66.0) | 44/78 (56.4) | 35/122 (28.7) |
| Disability insurance status |  |  |  |
| Unknown disability insurance status | 7/735 (1.0) | 0/78 (0.0) | 0/121 (0.0) |
| Has applied for disability insurance | 40/735 (5.4) | 6/78 (7.7) | 4/121 (3.3) |
| Does not receive disability insurance | 457/735 (62.2) | 36/78 (46.2) | 59/121 (48.8) |
| Does receive disability insurance | 231/735 (31.4) | 36/78 (46.2) | 58/121 (47.9) |
| Has had a relapse in the past 6 months | 53/611 (8.7) | 7/62 (11.3) | 11/113 (9.7) |
| Uses a wheelchair | 94/739 (12.7) | 12/80 (15.0) | 35/122 (28.7) |
| Uses a cane or crutches | 108/739 (14.6) | 11/80 (13.8) | 36/122 (29.5) |
| Uses a rollator | 55/739 (7.4) | 7/80 (8.8) | 23/122 (18.9) |
| Able to use public transportation | 665/739 (90.0) | 70/80 (87.5) | 90/122 (73.8) |
| Swiss Citizen | 684/756 (90.5) | 72/85 (84.7) | 116/127 (91.3) |
| Living situation |  |  |  |
| Lives at clinic/nursing home | 1/728 (0.1) | 0/76 (0.0) | 2/120 (1.7) |
| Lives with family | 226/728 (31.0) | 25/76 (32.9) | 27/120 (22.5) |
| Lives with friends/relatives | 17/728 (2.3) | 0/76 (0.0) | 2/120 (1.7) |
| Lives alone | 151/728 (20.7) | 15/76 (19.7) | 31/120 (25.8) |
| Unknown living situation | 11/728 (1.5) | 0/76 (0.0) | 1/120 (0.8) |
| Other living situation | 7/728 (1.0) | 0/76 (0.0) | 0/120 (0.0) |
| Lives with parents | 15/728 (2.1) | 2/76 (2.6) | 2/120 (1.7) |
| Lives with spouse/partner | 300/728 (41.2) | 34/76 (44.7) | 55/120 (45.8) |
| Marital status |  |  |  |
| Divorced | 68/728 (9.3) | 12/76 (15.8) | 13/120 (10.8) |
| Married | 391/728 (53.7) | 33/76 (43.4) | 71/120 (59.2) |
| Unknown marital status | 15/728 (2.1) | 0/76 (0.0) | 2/120 (1.7) |
| In registered partnership | 8/728 (1.1) | 1/76 (1.3) | 2/120 (1.7) |
| Separated | 12/728 (1.6) | 0/76 (0.0) | 2/120 (1.7) |
| Unmarried | 220/728 (30.2) | 30/76 (39.5) | 24/120 (20.0) |
| Widowed | 14/728 (1.9) | 0/76 (0.0) | 6/120 (5.0) |
| Highest attained education |  |  |  |
| Mandatory schooling | 1/728 (0.1) | 0/76 (0.0) | 0/120 (0.0) |
| Mandatory schooling completed | 19/728 (2.6) | 2/76 (2.6) | 9/120 (7.5) |
| Apprenticeship | 274/728 (37.6) | 34/76 (44.7) | 60/120 (50.0) |
| Highschool | 73/728 (10.0) | 8/76 (10.5) | 5/120 (4.2) |
| Higher professional education | 110/728 (15.1) | 8/76 (10.5) | 16/120 (13.3) |
| University degree | 224/728 (30.8) | 22/76 (28.9) | 21/120 (17.5) |
| Other education | 14/728 (1.9) | 1/76 (1.3) | 6/120 (5.0) |
| Unknown education | 13/728 (1.8) | 1/76 (1.3) | 3/120 (2.5) |
| Number of times person was physically active for at least 30 minutes in past 7 days |  |  |  |
| Not physically active | 98/726 (13.5) | 15/79 (19.0) | 27/121 (22.3) |
| Once per week | 74/726 (10.2) | 7/79 (8.9) | 15/121 (12.4) |
| Twice per week | 145/726 (20.0) | 11/79 (13.9) | 15/121 (12.4) |
| Three times per week | 123/726 (16.9) | 16/79 (20.3) | 18/121 (14.9) |
| Four times per week | 78/726 (10.7) | 5/79 (6.3) | 6/121 (5.0) |
| Five times per week | 94/726 (12.9) | 10/79 (12.7) | 15/121 (12.4) |
| Six times per week | 42/726 (5.8) | 4/79 (5.1) | 9/121 (7.4) |
| Daily | 72/726 (9.9) | 11/79 (13.9) | 16/121 (13.2) |
| EQ-5D index; N, median [interquartile range] | 744, 90.7 [81.5; 97.8] | 82, 83.2 [68.3; 92.0] | 125, 81.5 [64.9; 90.7] |
| Visual Analogue Scale; N, median [interquartile range] | 744, 80 [60; 90] | 80, 70 [50; 80] | 122, 70 [50; 80] |
| Number of self-reported symptoms; N, median [interquartile range] | 750, 3 [0; 7] | 81, 6 [2; 10] | 125, 6 [2; 9] |
| Electronic device use (***** indicates grouping variables) |  |  |  |
| At least weekly use of PC | 632 (81.9) | 70 (79.5) | 83 (63.8) |
| At least weekly use of smartphone* | 770 (99.7) | 83 (94.3) | 29 (22.3) |
| At least weekly use of smartwatch | 95 (12.3) | 10 (11.4) | 5 (3.8) |
| At least weekly use of tablets | 328 (42.5) | 36 (40.9) | 33 (25.4) |
| At least weekly use of apps***** | 718 (93.0) | 82 (93.2) | 21 (16.2) |
| At least weekly use of internet***** | 740 (95.9) | 86 (97.7) | 71 (54.6) |
| At least weekly use for verbal communication | 291 (37.7) | 51 (58.0) | 13 (10.0) |
| At least weekly use for written communication***** | 745 (96.5) | 83 (94.3) | 67 (51.5) |
| At least weekly use to make appointments***** | 131 (17.0) | 73 (83.0) | 11 (8.5) |
| At least weekly use to research health care providers***** | 3 (0.4) | 41 (46.6) | 2 (1.5) |
| At least weekly use to exchange with other persons with MS***** | 62 (8.0) | 40 (45.5) | 11 (8.5) |
| At least weekly use to retrieve general information | 445 (57.6) | 76 (86.4) | 41 (31.5) |
| At least weekly use to stay connected with health care providers***** | 3 (0.4) | 59 (67.0) | 2 (1.5) |
| At least weekly use to retrieve information on MS***** | 186 (24.1) | 69 (78.4) | 21 (16.2) |
| At least weekly use for self-tracking | 83 (10.8) | 31 (35.2) | 3 (2.3) |
| Would like to receive more support | 30 (3.9) | 12 (13.6) | 15 (11.5) |
