# Supplementary material for: Digital health for chronic disease management: An exploratory method to investigating technology adoption potential
Source: PLoS One. 2023 Apr 13;18(4):e0284477. doi: 10.1371/journal.pone.0284477 (PMC10101441; doi:10.1371/journal.pone.0284477)
Supplement: Attachment — Submitted filename: PLOS One Reviewrs comments.docx [file pone.0284477.s003.docx]

Digital health for chronic disease management: An exploratory method to investigating technology adoption potential

The study examined a low resource approach of capturing and clustering exposure and adoption of technology among patients with MS. The study has the possibility for a better characterization of prospective user populations and ultimately, future patient-facing technologies for broader audience.

However, the following concerns need to be addressed:

1. Numbering of in-text citation not clear, references numbering were not arranged in ascending order of citation . Authors should do necessary corrections following journals instructions
2. In line 136 authors should specify what the information from Swiss MS Registry (SMSR) was utilized for
3. In line 139; what is “the participation” referring to, is it your study or description of SMSR?
4. Under Materials and Methods subheading, what is the research design adopted in this study? Are there any other criteria for inclusion of participants in this study apart from age? Please provide details
5. In lines 141 to 146, authors should indicate specific duration of the longitudinal survey “semi-annual surveys” for how many years?
6. The extensive baseline survey is noted to contain information from short baseline survey. Please give more information on why this is needed in lines 146 to 148
7. Was the informed consent provided by participants written or oral? Line 149
8. Authors should provide a statement to clarify whether the SMSR survey released in October 2020 for capturing participants’ experiences during the SARS-CoV-2 pandemic was part of this study or an existing survey in the registry. Lines 152 to 153
